# The creation, implementation, and harmonisation of medical standard operating procedures and checklists of Finnish Helicopter Emergency Medical Service units

**DOI:** 10.1186/s13049-024-01241-x

**Published:** 2024-08-01

**Authors:** Simo Tukia, Jari Pirnes, Jouni Nurmi, Hilla Nordquist

**Affiliations:** 1 Emergency Medical Services, Wellbeing services county of Lapland, HEMS Unit FinnHEMS 51, Lentoasemankuja 18, Napapiiri, 96930 Finland; 2 Emergency Medical Services, Wellbeing Services County of Lapland, HEMS Unit FinnHEMS 50, Kauppakatu 25, Kemi, 94100 Finland; 3 Emergency Medicine and Services, Helsinki University Hospital and University of Helsinki, HEMS Unit FinnHEMS 10, Vesikuja 9, Vantaa, 01530 Finland; 4 FinnHEMS Oy, c/o Avia Pilot, Lentäjäntie 3, Vantaa, 01530 Finland; 5 Department of Healthcare and Emergency Care, South-Eastern Finland University of Applied Sciences, Pääskysentie 1, Kotka, 48220 Finland

**Keywords:** Standard operating procedures, Checklist, Helicopter Emergency Medical Services (HEMS)

## Abstract

**Introduction:**

The purpose of this study was to investigate the creation, implementation, and harmonisation of medical Standard Operating Procedures (SOP) in Finnish Helicopter Emergency Medical Services (HEMS). The research questions are: (1) What factors influence the creation and implementation of medical SOPs for Finnish HEMS units? and (2) What can be done to harmonise the medical SOPs of Finnish HEMS units?

**Methods:**

The research was conducted as a qualitative interview study with HEMS physicians who worked full-time in Finnish HEMS units or had worked in HEMS for more than five years. Three HEMS physicians from each of the six HEMS units in Finland participated in the study (*n* = 18). The thematic interviews (average duration 32 min) were transcribed (70,176 words in Finnish) and analysed using inductive content analysis.

**Results:**

The results of the first research question formed three main categories: (1) Background to developing medical SOPs and checklists (CLs), (2) Creation of medical SOPs in Finnish HEMS units, and (3) Implementation of medical SOPs and CLs. The main categories were divided into eight upper categories and twelve subcategories. The results of the second research question formed four main categories: (1) Prerequisites for harmonising procedures, (2) System-level changes needed, (3) Integrating common medical SOPs into HEMS, and (4) Cultural change. The main categories were divided into nine upper categories and nine subcategories.

**Conclusions:**

Medical SOPs and CLs are an integral part of Finnish HEMS. Each unit creates its own SOPs and CLs; their development, implementation, and follow-up are relatively unstructured. Harmonising existing SOPs would be possible, but developing common SOPs would require structural changes in HEMS and a stronger sense of community belonging among HEMS physicians.

## Background

Standard Operating Procedures (SOPs) are integral to many high-risk fields, such as the nuclear industry and aviation. They are detailed written instructions designed to standardise operations in a particular task [1]. Similarly, checklists (CL) are list-like tools that systematically note the action points of tasks and put them in order [2]. The beneficial effect of medical SOPs on patient safety has been proven in healthcare. For example, the World Health Organization’s (WHO) Surgical Safety Checklist, the best-known healthcare CL used globally, reduced postoperative complications and mortality by an average of 36% [3].Emergency medical services (EMS) have increasingly started to use SOPs in the treatment of various diseases [4]. SOPs have also been studied in Helicopter Emergency Medical Services (HEMS) units [5, 6].

Previous studies have demonstrated that SOPs have a positive impact on both individual procedures and on a general scale [4, 5, 7, 8]. In Hungary, seven HEMS units implemented an SOP for airway management, increasing the first-attempt intubation success rate from 68 to 95.4% after standardisation [5]. In Germany, three different CLs were developed and implemented in the EMS, significantly improving the quality of care in patient assessment and illness treatment [4]. A German study on the use of SOPs in emergency care for acute coronary syndrome found that while SOPs improved drug treatment, they did not significantly affect patient follow-up [7]. A study conducted in the Nordic countries investigated the effect of a CL as a tool for airway management in medical intubations performed by anaesthesiologists during emergency care. The use of the CL significantly improved the success rates of the first-attempt (96.6% vs. 86.2%) and second-attempt (99.4% vs. 95.7%) medical intubations. Oesophageal intubations were more common in the non-CL group (2.2% vs. 0.3%). There were no other adverse event correlations with CL use. Using the CL increased on-site time by several minutes (23.6 min vs. 27.5 min), and its usage varied widely among Nordic anaesthesiologists [9].

SOPs have also been studied between different operational units. For example, a study on the use of CLs in United Kingdom (UK) medical units showed that CLs are widely used but implemented in different ways. Some CLs were simple, while others were complex, with significant variations in content, language, and length [10]. A study on preoxygenation methods in all the HEMS units in the UK showed that all units’ SOPs included various oxygenation methods with significant differences in content. For example, preoxygenation was mandatory in 81% of the SOPs, while oxygenation during apnoea was required in 38% [6]. Evidence shows that identical SOPs and CLs can be successfully implemented across multiple operational units. For example, a study conducted in Los Angeles on the statewide implementation of an SOP to direct stroke patients to the appropriate treatment facility [8] showed that SOPs can also be successfully adopted at the state level.

SOPs and CLs are widely used in Finnish HEMS units; however, no common SOPs or CLs are currently in use across the units. Common SOPs and CLs would harmonise Finnish HEMS operations and, for example, facilitate the mobility of physicians between units. The creation and implementation of SOPs and CLs and their harmonisation have not previously been studied. The aim of this study was to investigate the factors influencing the implementation of common medical SOPs for Finnish HEMS units. The research questions are: (1) What factors influence the creation and implementation of medical SOPs for Finnish HEMS units? and (2) What can be done to harmonise the medical SOPs of Finnish HEMS units?

## Materials and methods

The study was conducted as a qualitative interview study, as there was a desire to explore the opinions and perspectives of HEMS physicians [11]. The consolidated criteria for reporting qualitative research (COREQ) [12] were used to support the research integrity [11].

The target group were HEMS physicians who worked full-time in Finnish HEMS units or had worked in HEMS for more than five years. The non-profit state-owned organisation FinnHEMS Oy operates medical helicopter operations in Finland. It organises HEMS operations at seven locations, six of which fly with a HEMS physician (Kuopio, Oulu, Seinäjoki, Tampere, Turku, and Vantaa). The Rovaniemi unit is staffed with two paramedics instead of a HEMS physician. FinnHEMS Oy oversees each base (hereinafter unit), including the staff (pilot and HEMS crew member) and the helicopter and ground units operate around the clock. The HEMS physicians are employees of different wellbeing services counties, which organise all health, social and rescue services in Finland. In this study, we focused only on those six units that employ HEMS physicians.

### Interview guide

The data were collected in thematic interviews. The interview guide (Table 1) was developed in cooperation with the research group. Additional questions were asked during the interview to deepen the responses.

**Table 1:** Interview guide

Main themes	Explanatory questions
Background	Are you a full-time HEMS physician? How long have you worked in HEMS?
Medical SOPs and CLs in Finnish HEMS activities	What is your experience of medical SOPs and CLs in your work? How do you use medical SOPs or CLs in your work?
Creating medical SOPs in HEMS units	How do you think a high-quality medical SOP or CL is created? How have you created medical SOPs or CLs in your unit?
Implementing medical SOPs and allocating resources	How have medical SOPs and CLs been implemented in HEMS? What kind of resources have been allocated for implementing medical SOPs and CLs?
Harmonising medical SOPs and CLs for all HEMS units	Do you consider it possible that Finnish HEMS units would have common medical SOPs or CLs? Yes or no? Why? What ways can you identify what would allow units to use common medical SOPs and CLs in patient care in the future?

### Data gathering

In this study, the sampling was partly purposive and aimed to ensure a balanced representation [11] from different HEMS units. Focusing on experienced HEMS physicians was intended to maximise the richness and relevance of the collected data [11]. The persons in charge of the six HEMS units were contacted about participating in this study. They were sent a recruitment letter, which they forwarded to the unit’s HEMS physicians, who emailed the researcher (ST) of their willingness to participate in the study. The first three HEMS physicians to volunteer from each unit were chosen as participants of the study, comprising a total of 18 interviewees. None of them cancelled or withdrew from the interview. The interviews were conducted using Microsoft Teams, and recorded and automatically transcribed. During each interview, only the interviewer (ST) and the interviewee were present. The interviews lasted between 23 and 48 min (an average of 32 min) and there were no follow-up interviews. The transcripts of the interviews were reviewed for accuracy, and the errors were corrected. The transcripts contained a total of 70,176 words (in Finnish).

### Analysis

The transcribed data was analysed using inductive content analysis [13]. Each of the research questions was analysed separately. The first author (ST) familiarised himself with the data by reviewing and rereading the transcripts. After this, the sentences and paragraphs that answered the research questions, were marked in different colours on the transcripts. Then, the marked content was condensed without altering its meaning and extracted into a new Word document. Then, the data were grouped so that similar content was combined to form subcategories, which were then named based on their content. These subcategories were further grouped into upper categories, also named according to their content. Finally, these upper categories were grouped under the main categories. The categories form a hierarchical response to each research question.

The grouping was an iterative process in which the first author (ST) analysed the data, and the last author (HN) reviewed the analysis until both researchers agreed on the results. The significance of the results was examined and reviewed by the entire research group.

### Reflexivity statement

The study was designed collaboratively by the four-member research team, which also included two highly experienced HEMS physicians (JP, JN). This supported the formulation of research questions relevant to the HEMS environment, the choice of methodology, the appropriate selection of the target group, and the design of the interview themes. ST (the first author) conducted the interviews and analysed the data. He has approximately ten years of work experience as a HEMS paramedic at the Rovaniemi unit, which is staffed with two paramedics. No interviews were conducted with staff at the Rovaniemi unit for this reason. The flow of the interviews and the posing of additional questions were supported by the interviewer’s extensive work experience in the HEMS environment, as the shared professional language facilitated communication despite different professional roles. This may have also engendered trust during the interviews and allowed for rapid deepening into the topic. However, as this was his first interview study, it might have affected the precision of follow-up questions. ST received guidance for conducting the interviews and analysing the data from HN (last author), who has extensive experience in qualitative research and no personal, only research-focused, connection to the HEMS environment. This supervisory support also included reviewing the condensed content, which supports the comprehensiveness of the original data. Overall, the close collaboration of the two researchers with different backgrounds during the analysis process helped ensure that the results were based on the interview data rather than, for example, personal views of the topic.

All researchers remained aware of the importance of an inductive approach throughout the research process. Discussing the results with the entire research team supported the relevance and quality of the qualitative reporting and ensured relevance to the HEMS environment. JP, JN, and HN were not aware of the identities of the interviewees, which further supported the inductive approach by ensuring no individual’s opinions were given undue prominence over others. Overall, the roles of the researchers were considered to ensure the study’s trustworthiness, acknowledging the inherent influence of researchers in qualitative studies and their relationship with the subject being studied [11]. This influence has been attempted to be highlighted transparently. Still, keeping a reflexivity diary would have further helped to document researcher-driven choices made at different stages [11].

### Ethical considerations

Research permits for the study were applied for from all hospital districts or wellbeing services counties under which the HEMS units operate. Participation in the interviews was based on informed consent. Participation was voluntary, and the interview could be interrupted or cancelled without negative consequences. The interviews did not contain any psychologically stressful elements and did not involve any risk [14]. No personal data was processed after the interviews were conducted. The original interview data was handled only by ST and HN, who are not colleagues of the interviewees.

## Results

Three emergency physicians from all six HEMS units in Finland participated in the study (*n* = 18). The results section presents the findings of the inductive content analysis, capturing the perspectives of the HEMS physicians interviewed.

### Factors influencing the creation and implementation of medical SOPs for Finnish HEMS units

In answer to the first research question, “What factors influence the creation and implementation of medical SOPs for Finnish HEMS units?” three main categories were identified in the interview data: Background to developing medical SOPs and CLs, the creation of medical SOPs in Finnish HEMS units, and the implementation of medical SOPs and CLs. The main categories were divided into eight upper and twelve subcategories (Table 2).

**Table 2:** The creation and implementation of medical SOPs in Finnish HEMS units

Main category	Upper category	Subcategory
Background to developing medical SOPs and CLs	The need to develop	Internal needs of the unit External needs of the unit Other usage needs
	Underlying factors	Factors guiding development Work unit and area of operation Influence of hospitals
	Intended effects	Patient benefit Making the work of HEMS physicians easier Improved HEMS team performance Developing EMS
Creation of medical SOPs in Finnish HEMS units	Responsible party	Individual Working group
	Unstructured development process	
Implementation of medical SOPs and CLs	Unit-level implementation	
	Integration into EMS	
	Challenges	

### Main category: Background to developing medical SOPs and CLs

#### Upper category: The need to develop

HEMS physicians described that their units’ internal needs for SOPs arise when procedures do not function optimally in the field. The need also arises in response to complex issues, rare and critical procedures, and frequent hyperacute situations. Furthermore, the introduction of new equipment, efforts to enhance team operations, or problematic variations in physician practices from the crew’s perspective, often necessitate the development of SOPs.

The need to develop SOPs and CLs can be driven by external factors such as adverse events, near misses, new research evidence, or national treatment recommendations that require simplification for HEMS use. Units where SOPs and CLs were already in common usage created pressure for those who had not implemented standard procedures.

*“We started getting some pressure from there [the emergency medical field] as these were already in use in other parts of Finland.”* (Interviewee 10).

The interviewees described that the need for SOPs and CLs may also arise from their use as tools for learning, teaching, and memory aids.

#### Underlying factors

Scientific knowledge was seen as providing the basis for SOPs, in addition to national treatment recommendations. Experiences working with similar HEMS units in Finland and abroad, taking into consideration aviation regulations, have also influenced the use of SOPs and CLs.

Work experience in various hospitals and HEMS units, variations between units in the guidance accuracy of different issues, regional differences, and geographical landscape have contributed to the need to create SOPs and CLs. The difference in equipment might necessitate the development of a specific SOP or the modification of another unit’s SOP. Ready-made procedures were sometimes modified due to a desire to create their own version.

*“One thing might just be that you don’t want to take the same [procedure] as somewhere else. You want to make your own. I don’t know if it’s like some kind of pride or what it’s about. But it may be that you think that if something over there [XX] is done like that*,* then yes*,* we will do better*,* or yes*,* we’ll handle it a little differently […] it’s probably also human*,* you want to make it your own*,* that it has been made by you. Not directly taken from somewhere else […] I don’t know if pride is the right word or not.”* (Interviewee 14).

The interviewees recognised that hospitals play a significant role in different specialties. The regional operating practices, the hospital resources in the HEMS units’ operating areas, and the working cultures and individuals in local hospitals were seen as important to consider when developing SOPs.

#### Intended effects

The positive effects would be the reasons for developing SOPs and CLs. Quality of care, reduced errors, and appropriate functioning of equipment achieved through SOPs and CLs were seen as improving patient safety. However, it was also noted that a potential risk to patient safety exists if SOPs are followed too rigidly or if the procedures do not take into consideration the patient’s needs or the time window.

It was described that the use of SOPs and CLs could make decision-making easier, reduce deviations, and potentially prevent things from being forgotten. These, in turn, could lead to a reduced workload, enhanced occupational safety, a greater sense of security, and the feeling that their work is meaningful.

SOPs and CLs could improve the operations of the entire HEMS team. When using SOPs, the team’s composition would not matter because all members know how to act. The structure of SOPs improves and speeds up operations as all team members can identify potential errors and respond in an expected way.

Using SOPs has brought about an overall positive development in the EMS. Operations become standardised and more predictable, even when working with an external or different team.

### Creation of medical SOPs in Finnish HEMS units

#### Responsible party

There is no designated person responsible for SOPs and CLs in the units. However, the responsibility is usually assigned to someone due to their qualifications or in relation to their other duties. It was seen as necessary that the person responsible for drafting SOPs and CLs had worked full-time in the HEMS unit to be sufficiently familiar with EMS and its requirements. Usually, other HEMS physicians would also review the draft SOP or CL at some point in the process.

*“Well*,* maybe something smaller like that*,* so some people become responsible for developing SOPs*,* and then it begins with them gathering information and research for that unit and then making some kind of operating model. Then these are discussed in our unit meetings*,* and then we try to refine it a bit more”* (Interviewee 2).

Informational sharing regarding different SOPs and CLs in use among HEMS units has increased, and the cooperation between units has improved. However, cooperation was seen as mainly taking place between the physicians in charge, and the interviews revealed differing opinions on whether this cooperation was adequate or not.

It was seen that the expertise of the HEMS physicians, crew members, pilots and specialists working in hospitals should be extensively utilised when developing SOPs and CLs. Multiprofessional and participatory development was also seen as mitigating resistance to change in implementing procedures.

#### Unstructured development process

The process for developing SOPs and CLs often lacks clarity. The time allocated to standardise a method ranges from a few hours to several months, depending on the unit. Development methods and timing can differ greatly between and within units, depending on the procedure type and scope. Effective early communication and a precise implementation timetable were seen as critical, yet often challenging to achieve in practice.

The EMS physicians emphasised the importance of collecting feedback and testing SOPs and CLs before implementation, using methods like simulation training, skill workshops, and pilot phases to assess and refine the procedures. After implementing an SOP or CL, its functionality should be assessed during regularly scheduled unit days for potential further development. While some procedures are reviewed annually and have a designated responsible person, not all SOPs or CLs are consistently monitored. It was seen as crucial to measure the outcomes of newly implemented procedures or CLs to identify potential benefits and assess HEMS crew members’ adherence to them.

*“That kind of retrospective systematic evaluation*,* in my opinion*,* occurs very rarely in the Finnish health care system*,* and it certainly does not occur very systematically in helicopter operations. I don’t know about other units*,* but for us to look and evaluate how this has gone*,* it’s not really done very systematically.”* (Interviewee 13).

### Implementation of medical SOPs and CLs

#### Unit-level implementation

The interviewees noted that the scope of the SOP and the available resources influence their implementation, leading to variation between and within units. Further, not all SOPs require an involved design and implementation process.

The need for well-planned communication was highlighted. Currently, the information on new SOPs and CLs is shared during unit or training days, via email and WhatsApp, followed by personnel training through videos, PowerPoint briefings, simulations, and animal or cadaver sessions. For some procedures, the training responsibility falls to individual HEMS physicians; however, there may not always be the possibility to train people adequately, often for valid reasons. HEMS physicians noted that insufficient training could affect the commitment to new procedures, which is why a slow and controlled introduction is often beneficial.

#### Integration into EMS

The training of other EMS professionals divided the interviewees’ opinions. On one hand, training was generally seen as important, and it was noted that other EMS fields already use SOPs. On the other hand, some SOPs were seen as specific to HEMS units, making broader EMS training unnecessary.

#### Challenges

A lack of commitment was identified as the biggest challenge in adopting SOPs. Implementation requires active discussion during the development process to identify and address obstacles. Moreover, insufficient training resources were seen as hindering commitment to the new practice(s). Poor implementation of CLs could result in a perceived increase in workload. Other identified challenges included the physical placement of SOPs/CLs, modifications by different physicians, and training part-time HEMS physicians.

### Harmonising medical SOPs of HEMS units

In answer to the second research question, “What can be done to harmonise the medical SOPs of Finnish HEMS units?” four main categories were identified in interview data: Prerequisites for harmonising procedures, System-level changes needed, Integrating common medical SOPs into HEMS, and Cultural change. The main categories were divided into nine upper categories and nine subcategories (Table 3).

**Table 3:** Harmonising medical SOPs and CLs in Finnish HEMS operations

Main category	Upper category	Subcategory
Prerequisites for harmonising procedures	Learning from the current situation	Existing procedures Harmonisation of the division of duties between units Strengthening competence to find a uniform structure
	National treatment recommendations for HEMS operations	
System-level changes needed	Harmonising EMS	One organisation in HEMS operations Medical management of HEMS operations
	Allocated resources	Human resources Equipment resources
Integrating common medical SOPs into HEMS	Increased collaboration	Balanced cooperation between units The influence of opinions and preferences
	Working groups	
	Training	
Cultural change	Desire to harmonise medical SOPs	
	Unit equality	

### Prerequisites for harmonising procedures

#### Learning from the current situation

Interviewees emphasised the need for mapping and harmonising existing SOPs or selecting the most suitable common SOP to take into shared use. A shared system could make all SOPs visible to all units, acting as an instruction matrix. SOPs should be adaptable to any unit, irrespective of their operational area. Understanding the differences in current practices could be achieved, for example, by increasing cooperation at the individual level between different units and through physicians’ unit rotation.

*“The practical division of duties has been agreed upon*,* it determines what a HEMS crew member does*,* what the doctor does*,* and so on*,* so on. It can also vary somewhat from unit to unit*,* as well as what those practices or notions are unless they are brainstormed and harmonised.”* (Interviewee 8).

The need to strengthen competence in developing SOPs and CLs included understanding the hierarchical and stylistic structure of SOPs to meet operational needs.

#### National treatment recommendations for HEMS operations

As a basis for developing SOPs, the interviewees proposed creating separate treatment recommendations for HEMS operations, which would provide general guidelines for out-of-hospital treatment. Common SOPs should generally be universal and specific, including only the essential elements. Harmonisation should first concentrate on medical procedures, although it was also seen as necessary in matters related to care and transport.

### System-level changes needed

#### Harmonising EMS

It was noted that harmonising SOPs would require a change in administrative management at the entire advanced care level. Regardless of the national emergency care system, transferring all medical activities under a single organisation would provide better support for the harmonisation of SOPs.

#### Allocated resources

According to the interviewees, the units would need enough full-time personnel to enable the development and harmonisation of SOPs. Common SOPs could require the standardisation of equipment between units.

### Integrating common medical SOPs into HEMS

#### Increased collaboration

The interviewees wished for increased collaboration in the entire EMS field. Equal collaboration was also hoped for at the unit level so that some units would not monopolise the development of SOPs. It was felt that there was not enough collaboration at the individual level, as an attachment to one’s own ways of working could form a psychological obstacle to harmonisation and adaptation.

*“Of course*,* it feels like*,* it’s always a bit like people have different ways of doing things*,* so how can we achieve this? Whose way is chosen [harmonised] without it always sparking discord.”* (Interviewee 5).

#### Working groups

The interviewees described that harmonised HEMS SOPs would need to be developed in a working group with equal representation from each unit. There was no consensus on the composition of the working group, but the leadership responsibility was partly assigned to the physicians in charge. Otherwise, it was hoped that the members would be enthusiastic and focused and that all HEMS physicians could participate in the development of SOPs.

#### Training

Standardised and combined training between units was seen as important for SOPs to be adopted and used consistently nationwide. This could be implemented with a dedicated training organisation, by units organising training, or by involving emergency physicians from different units. Allocating sufficient resources to arrange training was emphasised.

### Cultural change

#### Desire to harmonise medical SOPs

The need for everyone to be committed to the harmonisation of SOPs was emphasised. It was seen that units should operate uniformly nationwide, even if it requires individuals to deviate from their habits. However, some believe that SOPs should consider individual needs, such as equipment preferences.

#### Unit equality

HEMS units were felt to be in an unequal position, which caused friction and hindered the harmonisation of procedures. The interviewees explained that only a few HEMS physicians had worked in multiple Finnish HEMS units, resulting in a poor understanding of regional differences and individual unit needs. It was highlighted that HEMS physicians do not feel a wider sense of community, as each unit operates largely independently.

*“What we don’t have between FinnHEMS units is*,* it’s that sense of togetherness*,* and even though we all strive for the best possible result in terms of patient care*,* we’re missing the feeling of everyone at FinnHEMS working together.”* (Interviewee 15).

## Discussion

This is the first study to investigate the creation, implementation, and harmonisation of medical SOPs and CLs for Finnish HEMS units. The results show that SOPs and CLs have been developed and are widely used in Finnish HEMS operations. Moreover, the interviews revealed that prerequisites and the desire to harmonise procedures for all HEMS units currently exist. The results identified several existing factors that influence the creation, development and implementation of SOPs and CLs. Further, regarding the harmonisation of SOPs and CLs for all HEMS units, the results revealed the prerequisite conditions and potential methods to integrate common SOPs into all Finnish HEMS activities, along with the necessary system level and cultural changes.

According to the results, SOPs and CLs are already integral to Finnish HEMS operations, which is consistent with some high-risk sectors [1]. As a rule, HEMS physicians were very positive about SOPs and CLs, and they were considered important for both patient safety and their own work performance. Similar benefits of SOPs have also been observed in hospital emergency departments [15].

The results show substantial regional variation in developing SOPs and CLs in Finnish HEMS, and internal variation may also exist within units. There were variations, for example, in how comprehensively operations should be standardised. According to previous research, CLs developed individually on a unit can differ substantially from each other [6], which partially justifies the development of common SOPs and CLs. This study also suggested that SOPs, as a concept, can be understood in very different ways. The results show that SOPs and CLs for HEMS are still developing nationwide, and there is no clear consensus on where and how they should be developed. Internationally, the impact of SOPs or CLs on airway management has been studied extensively [5, 6, 9, 10, 16–18], and their use in HEMS is very common. In addition, research has been conducted, for example, on standardising the treatment of various diseases, ventilator care, and emergency driving [1, 4, 7, 8, 19–21]. However, according to the results of this study, CLs are often developed reactively in Finnish HEMS operations, for example, due to the procurement of new equipment or external pressure. Future research should investigate when it would be beneficial to develop SOPs and CLs in HEMS operations.

The results revealed significant differences in the implementation of SOPs and CLs. However, the interviewees generally felt that not all SOPs and CLs needed such an involved design and implementation process. On the other hand, it was clear that HEMS physicians would not commit to using SOPs and CLs if the implementation was not sufficiently resourced. Previous research supports this, as Olvera et al. (2022) showed that high-quality training is linked to how committed HEMS staff are to using CLs [22]. Post-implementation monitoring was also an issue that emerged in our results, as the regular monitoring of their effectiveness was only done for individual SOPs after their introduction. The high-quality implementation and monitoring of procedures were considered very important, but according to the results, these would require more resources. Previous implementation studies concerning extensive healthcare operating models have also emphasised the long-term nature of the process and the importance of continuous monitoring [23, 24].

According to the results of this study, there was a common desire among HEMS physicians for the harmonisation of SOPs for all HEMS units, for which the prerequisites already exist. This would be possible, for example, by examining the current SOPs already in place in each unit and identifying the possibilities for harmonisation. One of the biggest obstacles was the differences in the units’ operating areas, which are not sufficiently known outside the individual units. However, according to studies on airway management in Hungary and the United States, evidence suggests that it is possible to implement common SOPs to improve operations in several units [5, 22]. According to the results of this study, harmonisation can require operational changes at the individual level, at least partly, in which case individual HEMS physicians must change their usual practices and commit to the jointly agreed upon procedures. Harmonisation requires a cultural and structural change. Previous studies have found that changes initiated by professionals were the easiest to implement and resulted in the least resistance [25]; however, further research is needed to develop a culture that supports change at a strategic level.

The HEMS units involved in this research all have their own specific historical operational context, which was reflected in the results, for example, in the division of the field of HEMS physicians into northern and southern units. Cooperation between the physicians in charge of the units is constantly evolving, but it is possible that the units will still operate individually nationwide. Further studies should investigate what kind of system and cooperation structure would foster the unity of the entire HEMS physician profession and support the implementation of SOPs. In this study, many interviewees hoped to develop a national training system that would increase the frequency of interactions between HEMS physicians and help harmonise operations.

### Limitations of the study

This study was based on voluntary interviews with three highly experienced HEMS physicians from all six HEMS units in Finland (*n* = 18). The saturation of interview data [11] was achieved, which supports the assessment that the study produced an in-depth description of the phenomenon regarding HEMS operations in Finland. However, it should be noted that the HEMS physicians volunteering for the interviews might have had extensive personal experience and perspectives on the topic, meaning the results may not represent the views of all Finnish HEMS physicians and, for example, random sampling could have produced different results. Additionally, it cannot be completely ruled out that participants provided socially desirable responses or expressed opinions contrary to their true beliefs. However, no such indications were observed during the interviews. It is also possible that some relevant follow-up questions were not asked. Moreover, during the interviews, there were indications that the concept of SOPs is understood somewhat differently among HEMS physicians. In this case, the responses may have been about a broader whole than just the SOP itself, which may weaken the trustworthiness of the results. When this occurred, the interviewer (ST) clarified the concept of an SOP and CL.

The research process of this study has been carefully documented, and direct quotes from the original data have been given to verify the results [11]. The credibility of the results could have been strengthened by using different data collection methods [11] and long-term monitoring and participant reviews, meaning that the research findings would be compared with the participants’ original views [13, 26], but this was not possible due to the research design and resources. The dependability of the analysis [13, 26] was strengthened by the collaboration between the researchers, in which the first author carried out the data analysis and the last author checked the accuracy throughout the research process, and the final results were reviewed by the entire multidisciplinary, four-member research group.

Regarding the overall trustworthiness and transferability of results [13, 26], it should be noted that in this study, attitudes towards the harmonisation of SOPs and CLs between units were quite positive, although partly critical, too. Differences in EMS systems can affect the transferability of results [13, 26], which may limit, for example, their international usability. However, generalisation was not sought in line with the qualitative approach [11], but the results encourage further research in Finnish and other EMS contexts.

## Conclusions

Medical SOPs and CLs have been developed and are widely used in Finnish HEMS and are developed on a unit-by-unit basis. They are considered to be very important for work performance, consistent quality, and patient safety. The results reveal that the reasons behind the development of SOPs and CLs are often reactive rather than proactive. The high-quality implementation of SOPs and monitoring of their effectiveness would require additional resources. In addition, harmonising SOPs and CLs would require both structural and cultural changes to increase the physicians’ sense of belonging to a larger HEMS community.

Further studies should investigate what system and cooperation structure would better unite HEMS physicians and support the high-quality implementation of SOPs in operations in Finnish HEMS and other countries.

## Data Availability

The datasets generated during the current study are not publicly available.

## Declarations

### Author information

Simo Tukia is an experienced HEMS paramedic with a Master’s degree and previous research experience in a nationwide multicentre study of emergency medicine. Jari Pirnes is a physician and has worked in HEMS for over 16 years. He has previous research experience in HEMS and the challenges of emergency care in the Arctic environment. Jouni Nurmi is an adjunct professor and physician who has worked in HEMS for more than 10 years and has also done administration, research and teaching work related to the field. Hilla Nordquist is a principal lecturer and adjunct professor with a background in health sciences and disaster medicine. Her research interests have focused especially on the occupational wellbeing aspects of EMS personnel and the functioning of the EMS.

## Abbreviations

CL: Checklist
EMS: Emergency Medical Services
HEMS: Helicopter Emergency Medical Services
SOP: Standard Operating Procedures
UK: United Kingdom
WHO: World Health Organization
